# Ultrafast Laser Processing of Nanostructured Patterns for the Control of Cell Adhesion and Migration on Titanium Alloy

**DOI:** 10.3390/nano10050864

**Published:** 2020-04-30

**Authors:** Antoine Klos, Xxx Sedao, Tatiana E. Itina, Clémentine Helfenstein-Didier, Christophe Donnet, Sylvie Peyroche, Laurence Vico, Alain Guignandon, Virginie Dumas

**Affiliations:** 1 SAINBIOSE Laboratory INSERM U1059, University of Lyon, Jean Monnet University, F-42270 Saint Priest en Jarez, France; antoine.klos@3sr-grenoble.fr (A.K.); sylvie.peyroche@univ-st-etienne.fr (S.P.); vico@univ-st-etienne.fr (L.V.); alain.guignandon@univ-st-etienne.fr (A.G.); 2 Hubert Curien Laboratory, University of Lyon, Jean Monnet University, UMR 5516 CNRS, F-42000 Saint-Etienne, France; xxx.sedao@univ-st-etienne.fr (X.S.); tatiana.itina@univ-st-etienne.fr (T.E.I.); christophe.donnet@univ-st-etienne.fr (C.D.); 3 GIE Manutech-USD, 20 rue Benoit Lauras, F-42000 Saint-Etienne, France; 4 Laboratory of Tribology and Systems Dynamics, National School of Engineers of Saint-Etienne, University of Lyon, UMR 5513 CNRS, F-42100 Saint-Etienne, France; clementine.didier@enise.fr

**Keywords:** femtosecond laser, multiscale-patterning, wettability, human mesenchymal stem cell, cell adhesion, cell spreading, cell motility, protein adsorption

## Abstract

Femtosecond laser texturing is a promising surface functionalization technology to improve the integration and durability of dental and orthopedic implants. Four different surface topographies were obtained on titanium-6aluminum-4vanadium plates by varying laser processing parameters and strategies: surfaces presenting nanostructures such as laser-induced periodic surface structures (LIPSS) and ‘spikes’, associated or not with more complex multiscale geometries combining micro-pits, nanostructures and stretches of polished areas. After sterilization by heat treatment, LIPSS and spikes were characterized to be highly hydrophobic, whereas the original polished surfaces remained hydrophilic. Human mesenchymal stem cells (hMSCs) grown on simple nanostructured surfaces were found to spread less with an increased motility (velocity, acceleration, tortuosity), while on the complex surfaces, hMSCs decreased their migration when approaching the micro-pits and preferentially positioned their nucleus inside them. Moreover, focal adhesions of hMSCs were notably located on polished zones rather than on neighboring nanostructured areas where the protein adsorption was lower. All these observations indicated that hMSCs were spatially controlled and mechanically strained by the laser-induced topographies. The nanoscale structures influence surface wettability and protein adsorption and thus influence focal adhesions formation and finally induce shape-based mechanical constraints on cells, known to promote osteogenic differentiation.

## 1. Introduction

Because of their excellent biocompatibility and superior mechanical strength, titanium and related alloys, such as titanium-6aluminum-4vanadium (Ti6Al4V), are widely used for biomedical applications [1,2]. However, it remains a challenge to achieve a homogenized bone–implant interface for decent osseointegration of dental and orthopedic implants in human bodies. Several attempts to solve this issue have been reported. In particular, it was shown to be possible to modify surface bio-functionalities, such as improved osseointegration at the implant surface, through surface engineering [3,4]. The process of osseointegration involves a complex chain of events, from protein adsorption to recruitment of mesenchymal stem cells (MSCs) and osteoblasts, finally leading to bone formation at the interface of implant.

Human MSCs (hMSCs) are multipotent bone-marrow derived stem cells that can differentiate in a wide diversity of cell types such as osteoblasts, adipocytes, chondrocytes or myoblasts and they represent a model of choice in the field of biointegration tissue–implants. It is widely accepted that surface properties and, in particular, topography and wettability affect protein adsorption and the subsequent cell adhesion [5,6] and fate [7]. Surface micropatterning can be applied to modify the microenvironment of cells and modulate their behavior. Most micropatterning techniques used in order to apply shape-based mechanical constraint on cells [8] derive from surface grafting of pro-adherent proteins surrounded by an anti-adhesion coating. Others (e.g., photolithography) are mostly applied to polymers [9]. Laser texturing is the only technique that can, in a single step, provide controlled multiscaled design on metallic surfaces. Previous studies showed that by inducing sub-micrometric roughness, laser texturing could enhance osteoblastic differentiation of hMSCs [10,11] and in vivo osseointegration [12,13]. Many efforts have been made in order to enhance the osteogenic potential but engraftment of hMSCs is a critical step in the process of osseointegration. Hence, ultrafast laser texturing appears to be a promising surface biofunctionalization approach to control hMSCs adhesion, spreading and migration. It was widely demonstrated that multiscale surfaces can be achieved by using femtosecond laser texturing, such as nano-textured grooves [14,15], grids [11] or pits [10] within the micrometric scale. Moreover, laser texturing allows one to obtain various types of laser-induced surface nanostructures, such as laser-induced periodic surface structures (LIPSS), grooves, spikes, columns, etc. [16,17,18,19,20,21]. Of peculiar bio-engineering importance, these nanostructures have different wetting properties [19,22], which are time-dependent and age towards hydrophobicity [23,24].

Here, our goal was to create finely designed patterns on a Ti6Al4V surface, with both micro- and nanometric dimensions. It is of interest to evaluate the influence of these engineered double-scaled surface patterns on cell behavior. It is known that hMSC osteoblastic differentiation could be directed by the state of intracellular tension [8,25,26,27,28]. In order to engineer titanium surfaces with optimal properties for improved osteogenesis, one must understand the complex interrelationships among material surface properties, adsorbed proteins, and early cellular responses. In this in vitro study, we focused on the short-term cellular responses which are known to be crucial in regulating events that lead to cell differentiation. The aim was to investigate the influence of the laser nanostructures on cell adhesion, spreading and migration and compare it to conventional smooth titanium. We analyzed the wetting properties of laser nanostructures before and after sterilization, then we investigated the effect on the adsorption of proteins on these areas and the establishment of focal contacts. The multi-scale complex design was conceived in order to spatially control cell adhesion and multicellular organization. Specific design with carefully arranged pro- and anti-adhesive areas could mechanically constrain the cellular morphology, which is critical in numerous biomedical and tissue-engineering applications.

We first investigated the properties, such as the topography and wettability, of surfaces fully covered by simple laser-induced nanostructures, and their influences on cell spreading and motility were also quantified. Based on the knowledge acquired from the aforementioned studies, we designed and laser-texturized multiscale surface patterns with combinations of nanostructures, micro-pits, and polished surface areas. Such surface patterns were developed in order to position cell nuclei and stimulate the cells to adopt a desired cell shape, thanks to anti-adhesive nanostructures. The key performance factors, such as the distribution of vinculin adhesion points, surface protein adsorption rates and the nuclei location were characterized.

## 2. Materials and Methods

### 2.1. Titanium Alloy Samples

Mirror-polished titanium alloy grade 5 (Ti6Al4V) samples were purchased from Goodfellow (Huntingdon, UK). The samples were square with dimensions of 10 mm by 10 mm with a thickness of 1 mm.

### 2.2. Laser Surface Texturing

Surface texturing and patterning were performed by a femtosecond laser with a galvanometer scanner system within the GIE Manutech-USD platform (Saint-Etienne, France). As a laser source, Tangor HP (Amplitude Systems) was used delivering femtosecond laser pulses with a duration of about 400 femtosecond (fs) at a wavelength of 1030 nm. After passing through a 56 mm f-theta lens, the laser beam converged to a focus spot with diameter 2ω_0_ = 16.3 µm (1/e^2^) in the focal plane. The laser fluence was adjusted by varying the laser power at a given laser repetition rate. The laser was linearly polarized.

Two different types of laser-processed surfaces were realized:

- two entirely laser-nanostructured samples fully covered by either LIPSS or spikes, termed as such in the following;
- two multiscale laser-patterned (LP) samples covered with a combination of micro-pits and laser-nanostructured patterns, together with stretches of polished surface areas (LIPSS + polished or spikes + polished), termed LP_LIPSS and LP_spikes, respectively.

LP_LIPSS and LP_spikes were realized through a multiple-step patterning strategy: the stretches of LIPSS or spikes areas were first laser-patterned and micro-pits were then machined in the next step. A spiral trajectory from the periphery to the center of the micro-pits was used to obtain a homogeneous photon energy distribution and, therefore, a homogeneous depth. A superposition of the features made up the complex surface patterns. The principle laser parameters for generating the aforementioned structures are summarized in Table 1. The laser-processed samples were then sonicated in demineralized water to clean them after which they were dried at room temperature.

### 2.3. Surface Topography

Topographies and roughness (*S*_a_) were analyzed using an optical 3D microscope (InfiniteFocus G4, ALICONA, Graz, Austria) which uses the focus variation microscopy technique. This allows 3D surface topography images to be obtained in which each pixel is at its maximum focus and from which the altitude variations can be analyzed. *S*_a_ (Arithmetical mean height of the surface) is a 3D-roughness parameter defined by the ISO 25178 standard. For further surface analysis, scanning electron microscopy (SEM) (TESCAN VEGA3 SB, Brno, Czech Republic) was carried out at 20.0 kV with a secondary electron detector.

### 2.4. Surface Wettability

Wettability measurements were carried out with a laboratory-developed multiscale and multifunctional system within the GIE MANUTECH-USD consortium. They were performed in a controlled atmosphere (Temperature (T) = 23 ± 0.6 °C, Relative humidity (RH) = 40 ± 4%). Cell basal medium was used as the testing medium (MSCBM, Prod. No. PT-3238, Lonza, Basel, Switzerland). Approximately 2-µL droplets of culture medium were deposited on the surfaces, and the evolution of the droplet shape was visualized with a camera and a sample rotation stage enabling 360° contact angle measurements. The platform moved at a speed of 0.1 rad·s^−1^. The droplet profile and especially the contact angle (CA) were extracted from a complete droplet 360° rotation leading to approximately 50 measurements per droplet. The first CA measurements were carried out 38 days after laser processing. The second step of CA measurements was performed on the same samples, 24 h after a sterilization procedure in which the samples received a dry heat treatment at 180 °C for 2 h. The data represent the measurements from 4 consecutive droplets on each surface.

### 2.5. Cell Culture

Human bone marrow-derived mesenchymal stem cells (Lonza) at passage 6 were maintained in a T75-flask for a week in a growth medium (MSCGM, Prod. No. PT-3001, Lonza) before seeding. Cells were seeded on samples at 4000 cells·cm^−2^ in 6-well plates for 24 h, except in the case of the adhesion plaques study for which the growth time was 48 h.

### 2.6. Fluorescent Cell Labeling

Seeded cells were fixed in 4% paraformaldehyde for 30 min. Permeabilization was performed with 0.1% Triton X-100 in phosphate-buffered saline (PBS) for 3 min. Samples were incubated with rhodamine-conjugated phalloidin diluted at 1:50 in PBS, at 37 °C for 1.5 h, then with vinculin antibody, FITC (Fluorescein isothiocyanate) conjugated (Prod. No. F7053, Sigma-Aldrich, St. Louis, MO, USA) diluted at 1:50 in PBS, at 4 °C for 12 h. Afterwards, nuclei labeling was performed with 1 μg·mL^−1^ DAPI (4′,6-diamidino-2-phenylindole) diluted in PBS at 37 °C for 20 min. Washes were performed using PBS between each step of the experiment. Specimens were observed by confocal microscopy (ZEISS LSM 800 Airyscan, Oberkochen, Germany).

### 2.7. Living-Cell Labeling

For living-cell observations, DIL (1′-dioctadecyl-3,3,3′,3′,3′-tetramethylindocarbocyanine perchlorate, Prod. No. D282, Thermofisher, Waltham, MA, USA) previously diluted at 1000 µM in DMSO (Dimethyl sulfoxide) was used. DIL has a high affinity for the phospholipids that make up the cellular membrane and becomes fluorescent when binding with them. Cells were labeled in suspension at 1:500 with the previous solution in growth medium, and incubated (temperature (T) = 37 °C, relative humidity (RH) = 95%, 5% CO_2_) for 1 h, following the protocol of Dumas et al. [10]. After centrifugation (5 min, 2000 rpm), the culture medium was changed. Then, DIL-labeled cells were seeded on samples at a density of 4000 cells·cm^−2^. After letting the cells adhere to the surface for 1 h, the samples were put upside-down on biocompatible silicon pillars before the time-lapse recording started. The confocal microscope (ZEISS LSM 800 Airyscan) was equipped with an incubator 37 °C, 5% CO_2_. Images were recorded every 30 min for 63 h.

### 2.8. Simultaneous Visualization of Surfaces and Cells through Confocal Microscopy

Surfaces and mesenchymal stem cells labelled with different fluorescent-coupled antibodies and fluorescent dyes were observed simultaneously using confocal microscopy. Labeling detection was performed by using up to three different laser wavelengths: 405 nm for DAPI, 488 nm for anti-vinculin-FITC antibody and 561 nm for rhodamine-conjugated phalloidin. Surfaces observation was based on the laser reflection on the substrate. For this, images were acquired in a 560–700 nm wavelength detection range, including the fourth laser emission wavelength at 640 nm, here used as a spotlight. This is why polished surfaces, which are highly reflective, appear brighter than laser-nanostructured areas.

### 2.9. Protein Adsorption Assay

This experiment was based on the work of Miao et al. [29]. FITC-albumin (Prod. No. A9771, Sigma–Aldrich) was dissolved in PBS at 15 µM and then diluted at 1:100 in basal culture medium. Afterwards, 70 µL of FITC-albumin solution was deposited on sample surfaces and incubated overnight at 4 °C. Samples were carefully rinsed in PBS before observation by fluorescent microscopy (ZEISS LSM 800 Airyscan). The albumin adsorption rate is related to the mean fluorescence intensity. In order to measure such intensity on specific zones, the laser-patterned surface was thresholded to identify polished and nanostructured areas. Thus, a Boolean operation allowed us to individually measure the mean fluorescence intensity of both polished and nanostructured areas. The difference between both intensities was then statistically compared to the theoretical value µ_0_ = 0, which represents the no intensity difference. The protein adsorption assay was made on sterilized samples to consider the surface properties changes.

### 2.10. Image Analysis for Cell Behavior

Images were analyzed using the ImageJ [30] freeware with Fiji [31] package.

Spreading measurements were obtained after automatic image processing including binarization preceded by 8-bit conversion, background removal and filtering. Measurements were carried out on 4 different areas (1.25 × 1.25 mm^2^) and in duplicate.

For cell motility analysis, the “Manual Tracking” plug-in and IBIDI′s plug-in “Chemotaxis and Migration Tool” (Gräfelfing, Germany) were used with ImageJ on contrast-adjusted videos. Tortuosity was defined as the ratio between the accumulated distance and the Euclidean distance. It was computed every 3 frames (1.5 h) and averaged on the whole recording for each cell. Cell velocity was defined from the ratio of the accumulated distance and the total recording duration, for each cell. Mean acceleration was computed from two consecutive cell velocity values, which were calculated from 2 consecutive cell position measurements within a time step between frames of 30 min. The absolute value of acceleration/de-acceleration was then averaged cell by cell for the whole migration path (63 h).

Nuclei location measurements were performed after binarization preceded by 8-bit conversion, background removal and filtering, with an automatic image processing for the counting of the whole nuclei. The counting of nuclei inside micro-pits was done manually, considering a nucleus inside a pit when half or more of the projected surface of the nucleus was inside. Data corresponding to 300 nuclei analyzed for LP_LIPSS and 494 for LP_spikes was normalized according to the surface proportion of micro-pits.

The proportion of focal adhesion points on polished surfaces was measured with consideration taken to both their number and their surface. The number of focal adhesion points was determined manually whereas the measurement by surface was semi-automatic. Indeed, focal adhesion points were binarized after an image processing following the steps: (i) coarse blurring (Gaussian, radius = 40); (ii) successive subtraction of the blurred image to the raw image followed by local contrast enhancement; (iii) successive slight blurring (median, radius = 1) followed by local contrast enhancement. Moreover, the laser-patterned surface was filtered to identify polished and nanostructured areas. Then, a Boolean operation was used to only measure the surface of focal adhesion points on polished areas. Data were normalized according to the proportion of polished areas.

### 2.11. Statistical Analysis

Data were analyzed through the Rstudio [32] freeware. Shapiro–Wilk normality tests and Bartlett homoscedasticity tests were performed for following statistical test selection. The Kruskal–Wallis test followed by a Mann–Whitney pairwise comparison with Bonferroni correction was used for statistical analysis (*n* < 15, or homoscedasticity or normality of data not respected).

## 3. Results

### 3.1. Surface Characterization

#### 3.1.1. Multiscale Surface Topography

**Figure 1:**
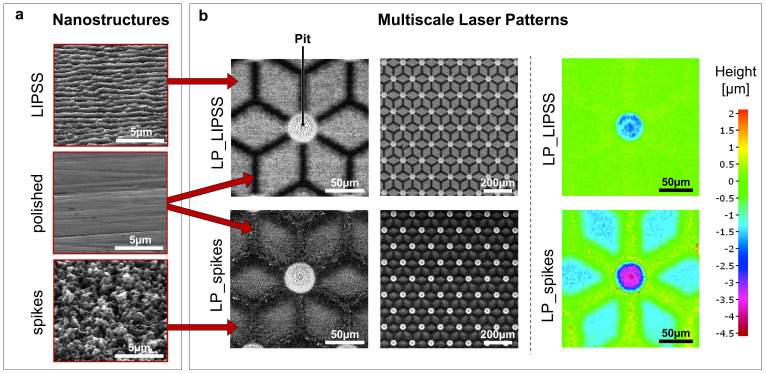
Nanostructures (LIPSS or spikes) and multiscale laser patterns (LP) produced by a femtosecond laser. (**a**) Scanning electron microscope (SEM) micrographs of polished surfaces and laser-nanostructured surfaces with LIPSS or spikes (tilt 45°). (**b**) SEM micrographs and focus variation microscopy of laser-patterned surfaces. LP_LIPSS and LP_spikes with a mix of polished and nanostructured areas. The micro-pits located in the center of the patterns were deeper and covered with LIPSS.

Two simple laser-induced nanostructures based on a literature survey [16,17,19] were obtained by adjusting femtosecond laser parameters (Figure 1a left panel). LIPSS and spikes were chosen because of their relative differences in terms of topography, small roughness and hydrophobic behavior. LIPSS are, as their name suggests, periodic (about 600 nm) and highly anisotropic whereas spikes are poorly periodic. The average areal surface roughness (*S*_a_) was measured to be 76 ± 5 nm for the LIPSS and 85 ± 5 nm for the spikes, which is consistent with other studies [33,34]. The *S*_a_ of the polished surface was 65 ± 4 nm.

By adapting laser trajectories, multiscale laser-patterned surfaces can be created. The laser-untouched areas remain polished whereas their laser-processed counterparts become either textured with LIPSS or with spikes. Furthermore, finely increasing the number of laser scan passes and laser fluence allows an etching of the surface to several micrometers of depth. Hence, it is possible to obtain microscale surface modifications like laser-etched pits. Multiscale laser-patterned LP_LIPSS and LP_spikes surfaces (Figure 1) were thus formed first by microscale etching followed by imprinting of superficial laser-induced nanostructures. The patterns had a star-like shape with polished branches 60 µm long and 10 µm wide for an optimal adhesion of the focal contacts in these areas. The center of each pattern displayed pits approximately 3 µm deep and 40 µm in diameter. The geometry of the µm-pits was carefully designed for the nuclei to reside in. These parameters were the optimized values derived from a set of pre-selection experiments (data not shown). The distance between the micropatterns was adjusted to create a contiguous network of periodically repeated patterns. It is worth mentioning that, by using focus variation microscopy, we observed that the higher laser fluence used to generate spikes caused a difference in surface depth of about 1.5 µm relative to the polished areas (Figure 1b).

#### 3.1.2. Surface Wettability

**Figure 2:**
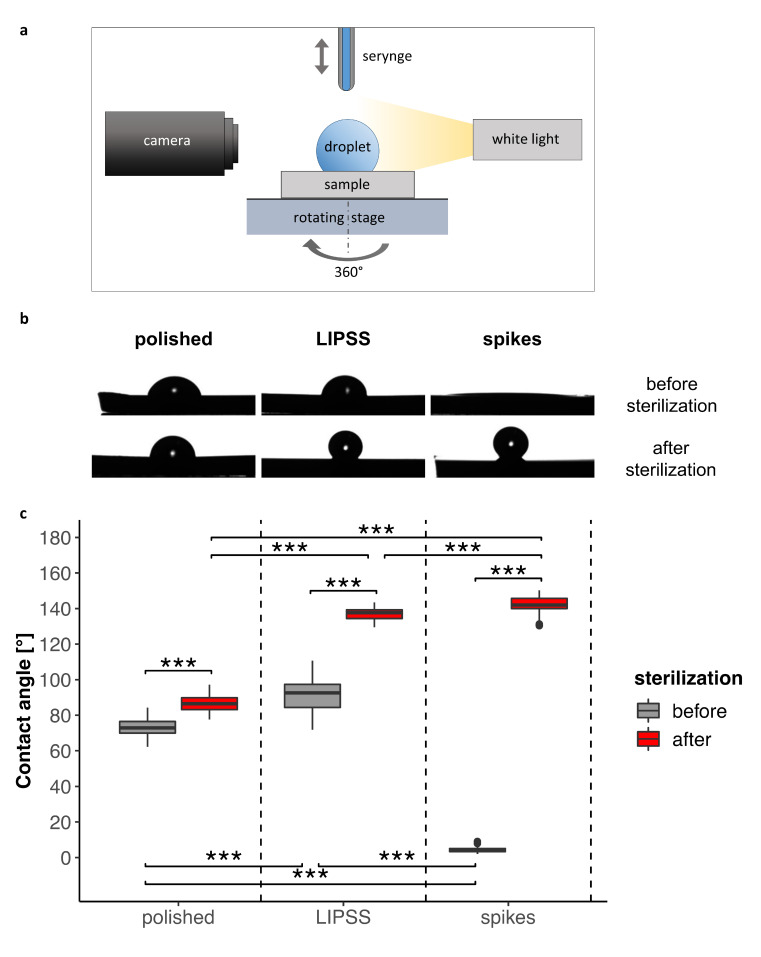
Comparison of the contact angles on polished and laser-nanostructured surfaces before and after sterilization. (**a**) Schematic of experimental setup for wettability characterization. (**b**) Representative droplet profile according to surfaces and sterilization process (**c**) 360° measurements from four consecutive droplets (at least 200 measurements per boxplot) of culture medium (2 µL) deposited on each surface (one sample). The Kruskal–Wallis test followed by Mann–Whitney pairwise comparison with Bonferroni correction was performed for data before and after sample sterilization. *** *p*-value < 0.001.

In order to characterize surface wettability by cell culture medium, contact angles were measured on both totally laser-nanostructured and polished surfaces. Figure 2 and Video S1 in Supplementary Materials show that contact angles were modified by laser texturing: they were either increased in the case of LIPSS, or notably decreased reaching the superhydrophilicity limit for spikes. It was observed that sterilization deeply affected the surface wettability. Indeed, we measured a significant increase in contact angle after such a treatment for all surfaces. This increase was remarkable for laser-nanostructured samples, reaching superhydrophobicity. The contact angle of the polished surface, however, did not change as much as the laser-nanostructured ones and remained within the same order of magnitude. Clearly, sterilization (180 °C, 2 h) made the nanostructured surfaces superhydrophobic while the smooth surfaces remained moderately hydrophilic.

### 3.2. Cell Behavior

#### 3.2.1. Cell Spreading

**Figure 3:**
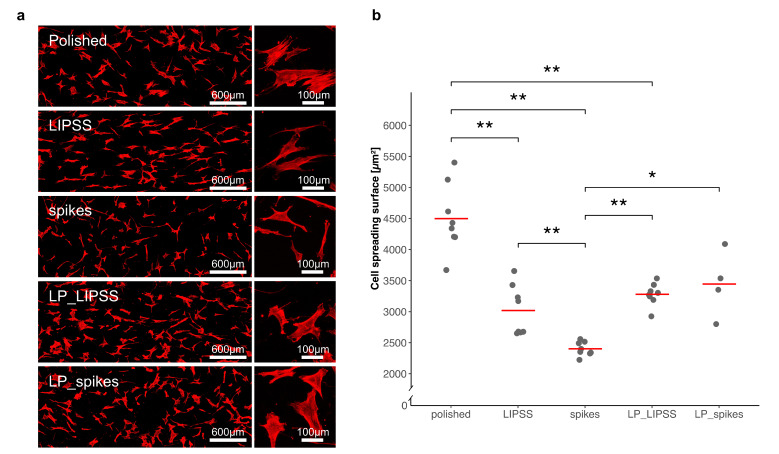
Quantification of cell spreading areas on polished, laser-nanostructured and laser-patterned surfaces. (**a**) Cytoskeleton staining images representing human mesenchymal stem cells (hMSCs) spreading 24 h after seeding on the five different surfaces previously sterilized. The rectangular graphs on the left are global views at a low magnification, and the square graphs on the right are representative cells at a high magnification; (**b**) four different areas of each surface were analyzed and, except for LP_spikes, the displayed data corresponds to duplicates of each condition (each dot represents the mean of more than 30 cells per area). The red lines denote mean values. The Kruskal–Wallis test followed by Mann–Whitney pairwise comparison with Bonferroni correction was performed. * *p*-value < 0.05, ** *p*-value < 0.01.

Cell spreading was investigated to quantify cell responses to nano-textured samples with different surface properties; 24 h after cell seeding, cytoskeletons and nuclei were stained to measure an average of cell areas. Figure 3 shows a significant decrease in cell spreading areas on laser-nanostructured surfaces compared with polished ones. Moreover, spreading on spikes occurred to a significantly smaller degree than spreading on LIPSS or LP_LIPSS and less by almost half compared with that on a polished surface. On LP_spikes, spreading increased compared with spikes, but did not reach the level observed on the polished specimens. Basically, cell spreading decreased in the presence of nanostructures (with or without smooth areas).

#### 3.2.2. Cell Motility

**Figure 4:**
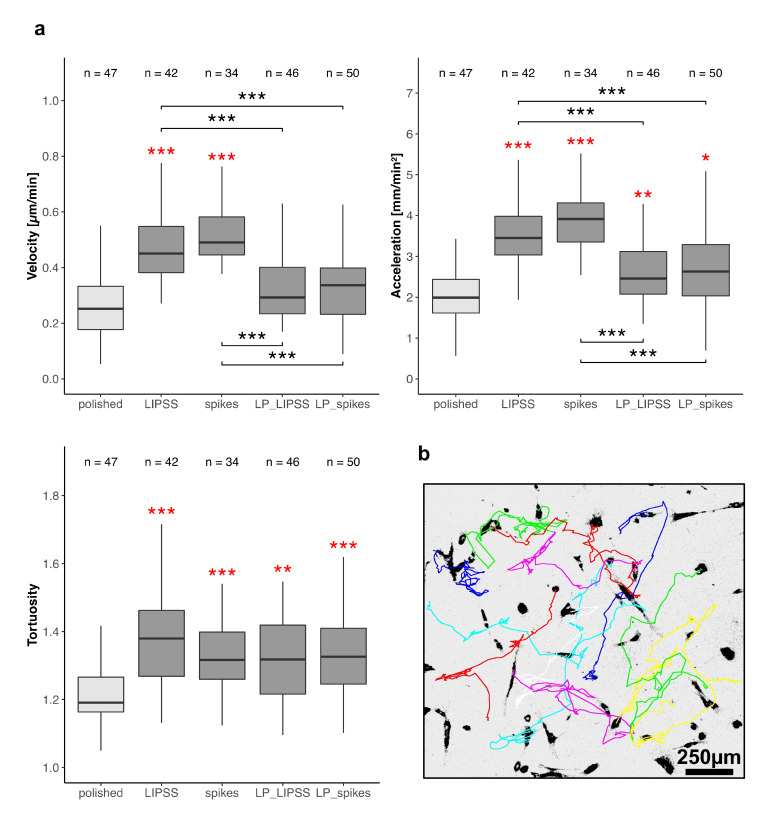
Cell motility on polished and laser-treated surfaces. hMSCs were observed 1 h post-seeding for 63 h and every 30 min on sterilized Ti6Al4V samples. (**a**) Mean velocity and acceleration (absolute value) were measured for each cell individually. Mean tortuosity was defined as the ratio between the accumulated distance and the Euclidean distance every 1.5 h and averaged on the whole record for each cell. Three areas per sample were analyzed, the exact number of analyzed cells is indicated above each boxplot. The Kruskal–Wallis test followed by Mann–Whitney pairwise comparison with Bonferroni correction was performed on data. * *p*-value < 0.05, ** *p*-value < 0.01, *** *p*-value < 0.001 Red asterisks denote significant differences versus polished surface. (**b**) Representative migration trajectories of cells on spikes by manual tracking of nuclei.

The cell velocity, acceleration and the tortuosity of trajectories were measured by manual tracking of nuclei (Figure 4b) on totally laser-nanostructured (LIPSS and spikes) and laser-patterned samples (LP_LIPSS and LP_spikes). The cell velocity was significantly increased (approximatively +100%) on LIPSS and spikes as compared with polished and laser-patterned surfaces (Figure 4a left panel). Furthermore, no difference in cell migration velocity appeared between polished and laser-patterned samples. Moreover, no cell velocity difference was noted between LIPSS and spikes nor between LP_LIPSS and LP_spikes patterns. The tortuosity of the trajectories was enhanced on all laser-treated surfaces compared with polished ones. Cell acceleration was increased on all the laser-treated samples whereas the mean velocity was only improved on LIPSS and spikes. This result strongly corroborated our observations from the time-lapse videos that the cells tended to stop their migration when crossing a micro-pit (see Video S2 in Supplementary Materials). Altogether, the results from a 63-h video strongly suggested that in presence of nanostructures the speed variations (cell acceleration or cell deceleration) increased and cell trajectories became more tortuous compared with the polished surface.

#### 3.2.3. Nuclei Location

**Figure 5:**
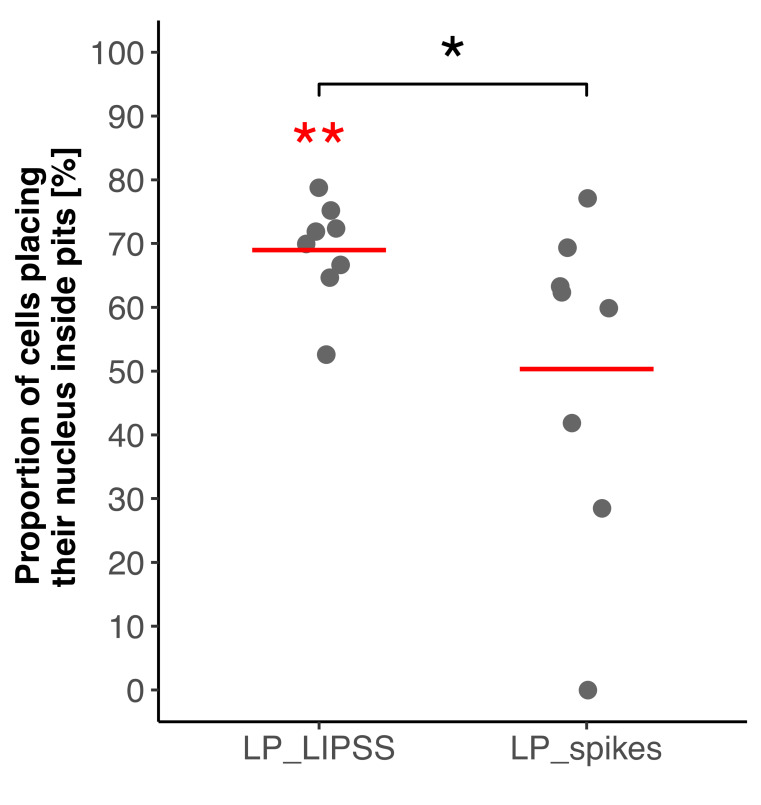
Quantification of nuclei proportion inside pits on laser-patterned surfaces 24 h after cell seeding. Values were normalized according to the proportion of pits area. Four different areas of each surface were analyzed and the displayed data correspond to duplicates of each condition (300 nuclei analyzed for LP_LIPSS and 494 for LP_spikes). Red lines denote mean values. The Mann–Whitney statistic test was performed. * *p*-value < 0.05, ** *p*-value < 0.01. Red asterisks denote significant differences with the random theoretical value (µ_0_ = 50%).

The nuclei location inside micro-pits was quantified. Figure 5 shows that the nuclei were preferentially located inside the micro-pits on LP_LIPSS surfaces, whereas this tendency was not observed on LP_spikes.

#### 3.2.4. Cell Adhesion

**Figure 6:**
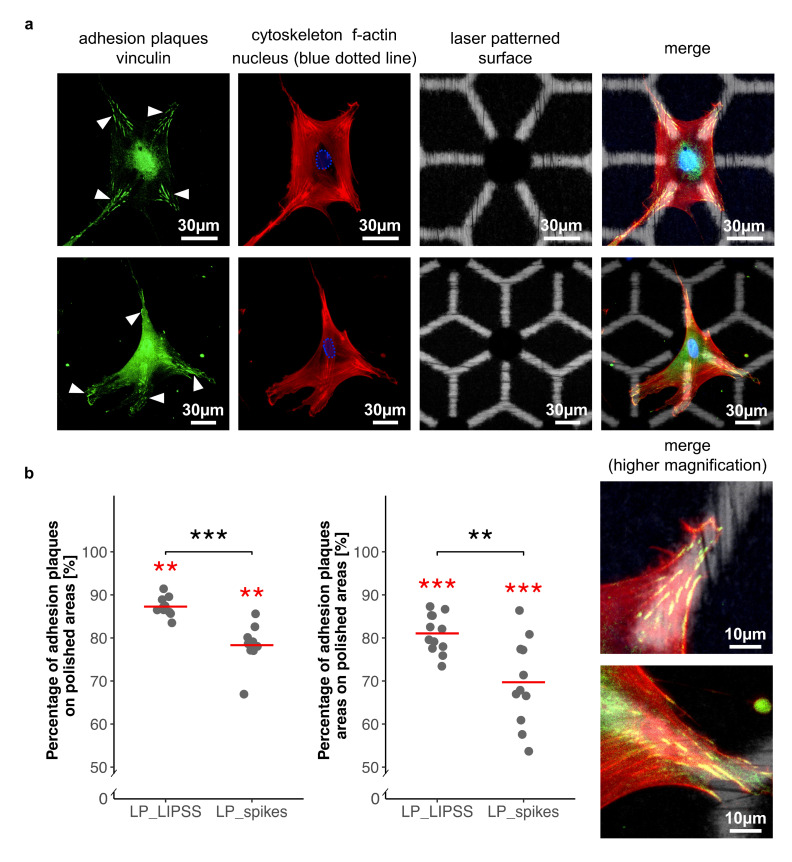
Quantification of focal adhesion repartition between polished areas and laser-nanostructured ones on laser-patterned surfaces. (**a**) Two representative cells 48 h after seeding on laser-patterned surfaces. Focal adhesions (white arrows) mostly located on polished areas. Nucleus in blue dotted line located inside the micro-pit. Cytoskeleton with a star like shape in red. Laser patterned surface with polished areas in white and nanostructured areas in black. (**b**) Ratio of adhesion plaques on polished regions compared to adhesion plaques on laser-nanostructured ones was obtained either by counting the number of adhesion plaques per cell (left panel) or by measuring the areas of the adhesion plaques per cell (right panel). A total of 760 focal adhesions were analyzed on LP_LIPSS and 665 on LP_spikes. Values were normalized according to the proportion of polished and laser-nanostructured regions on each sample. The Mann–Whitney U statistic tests were performed. Red lines denote mean values. ** *p*-value < 0.01, *** *p*-value < 0.001. Red asterisks denote significant differences versus the random theoretical value (µ_0_ = 50%).

In order to determine hMSC interactions with multiscale patterns, cell adhesion was investigated at the sub-cellular scale. For this, hMSCs were seeded on sterilized laser-patterned samples (LP_LIPSS and LP_spikes) and were analyzed by immunofluorescence assays for vinculin detection, a protein involved in cell adhesion. hMSCs were found to place significantly their focal adhesion points on polished areas rather than on laser-nanostructured ones (Figure 6a). These observations were confirmed by measurements after image processing (Figure 6b). The number and area of adhesion plaques were quantified, and data were normalized according to surface proportions. Both number and area measurements led to the finding that focal adhesions were mostly and preferentially located on the polished areas. Moreover, a significant difference was measured between laser-patterned LP_LIPSS and LP_spikes for both factors. Vinculin staining was also performed on polished samples, and samples fully covered by simple laser-induced nanostructures. It revealed that the focal adhesion clusters were well established on polished surface but no focal adhesion was observed on LIPSS or spikes (data not shown). This observation was consistent with the results concerning the preferential distribution of focal contacts on smooth areas of complex patterns.

Collectively, the migration versus adhesion data suggest that the complex patterns reduce the migration but increase the adhesion compared to simple nanostructures. This latter fact could be an important determinant for osteoblast differentiation.

### 3.3. Protein Adsorption

**Figure 7:**
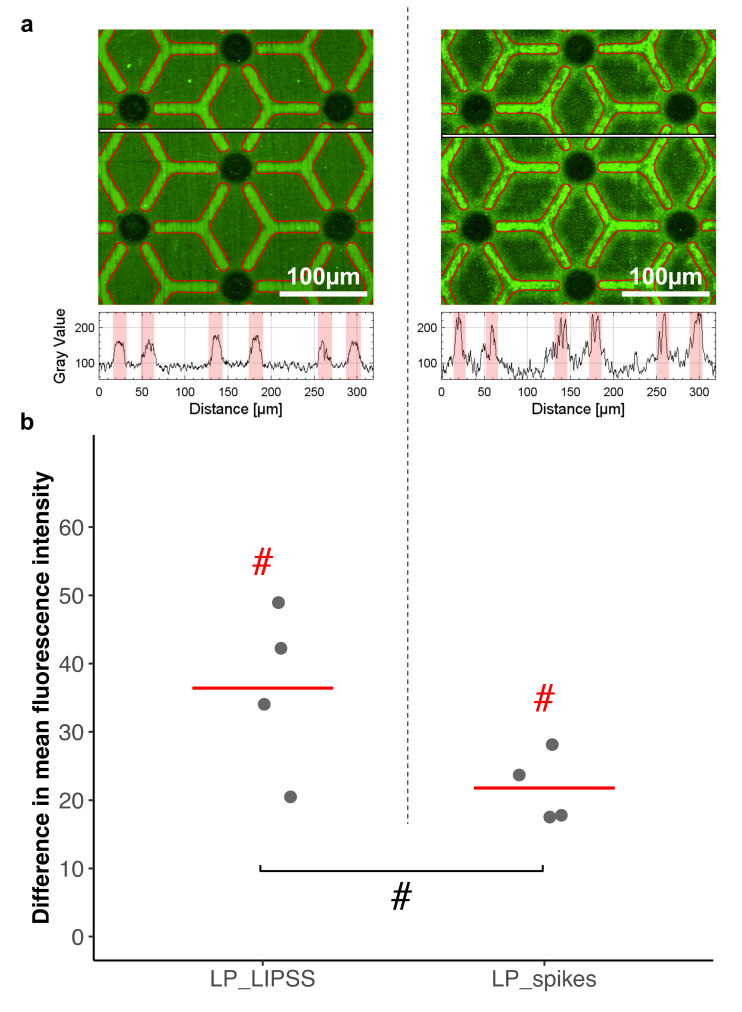
Comparison of albumin adsorption on polished areas and nanostructured areas. Fluorescent albumin was detected on laser-patterned surfaces with LIPSS (LP_LIPSS) or spikes (LP_spikes). (**a**) Representative image of fluorescence intensity. Polished areas are outlined in red, textured ones are the remainder (**top panel**). Respective profiles of the fluorescence intensity, red regions represent polished areas along the profile (**bottom panel**). (**b**) The difference in mean fluorescence intensity between polished and textured regions is displayed according to an 8-bit grayscale. Four measurements on areas of 320 µm by 320 µm per sample were analyzed. The Mann–Whitney one-tailed statistic test was performed. Red lines denote mean values. # *p*-value < 0.1. Red hash signs denote differences with the random theoretical value (µ_0_ = 0).

To observe preferential adsorption of protein an assay with fluorescent albumin was performed (Figure 7). During this experiment, laser-patterned samples LP_LIPSS and LP_spikes were investigated. The mean fluorescence intensity was measured on polished and on laser-nanostructured areas to quantify albumin adsorption on these surfaces. The mean fluorescence was found to be higher (*p*-value < 0.1) on polished areas than on their laser-nanostructured counterparts. It can be noted that the difference in mean fluorescence intensity was stronger for LP_LIPSS than on LP_spikes.

## 4. Discussion

### 4.1. Uniform Surface Topography (Polished Surface, Laser-Induced Periodic Surface Structures (LIPSS), and Spikes)

It is well known that the wettability of laser-nanostructured surfaces changes with time [23,24] and can be modified by storage in different media just after laser processing [35]. In fact, most laser-induced nanostructures show a total wetting (CA < 5°) shortly after the laser treatments. These structures then become more and more hydrophobic with the lapse of time. The first hydrophilic state could be explained by the laser removal of non-polar and organic surface contaminants [36]. Furthermore, according to the Wenzel model, the roughness of a hydrophilic surface enhances even more the hydrophilicity, hence the complete wetting observed on the sample with spikes. Moreover, it was shown that the laser irradiation increased the thickness of the natural oxide layer at the very surface of the laser-processed titanium samples [36,37] and this was especially demonstrated on LIPSS topographies. Such an oxide layer leads to high surface polarity, in turn related to hydrophilic properties due to the polarity of the water molecule [36]. More recently, surface aging towards hydrophobicity has also been shown to be connected with the progressive adsorption of non-polar and organic compounds [38]. It was proven that this trend could be accelerated through a low heat treatment (<200 °C) [37,39] comparable to the sterilization process used in this study.

Here, we show that total wetting could be reached in a relatively time-stable (38 days) manner by using laser-texturing (spikes). In particular, we emphasize that the sterilization process is a critical step for a more stable and reliable prediction of the surface properties for laser-nanostructured samples. Indeed, heat treatment appears to significantly promote hydrophobicity. The fact that these changes were not observed on polished surfaces contrary to their laser-nanostructured counterparts can be explained by the better-established chemical stability of the passivation layer of the polished surface. It remains challenging to analyze the independent effects of the surface topography and chemistry on wettability. Both are without doubt involved, but the cross effects are predominant. Because laser-induced nanostructures are generated by adjusting the laser fluence, a set of electronic, thermal and structural variations considerably affects the surface chemistry.

Some studies have already demonstrated the great influence of regular micro- and/or nano-structures on wettability [40,41] and that laser induced nanostructures such as LIPSS leads to anisotropy in wetting [19,42]. Hence, the large variation in contact angles on LIPSS (see the box plot in Figure 2) denoted a droplet anisotropy with an elongation perpendicular to the LIPSS waviness, which caused a wider contact angle range. The phenomenon became less evident after sterilization because of the shorter droplet baseline due to non-wetting. It is important to notice that a cell culture medium with an unknown surface tension was used as the testing medium for the contact angle measurements. This is why the results cannot be compared in absolute values with data from the literature; only relative trends can be proposed. This choice was made to mimic as well as possible real cellular experimental conditions.

Cells respond to their environment by adapting their behavior through changes in their morphology but also by adjusting their spreading, adhesion and motility. Such behavior modifications can influence cellular proliferation, direct cell fate, and trigger cellular differentiation, in particular when it comes to stem cells. Cellular morphology relies on the structure of the cytoskeleton, and changes in cell shape and contractility are, therefore, often a consequence of its (re)-organization. Moreover, cells display a mechano-sensing ability triggering the rearrangement of the cytoskeleton upon external mechanical stimuli. Thus, any morphological or mechanical deformation induced by the substrate topography are experienced by the intrinsic nuclear mechano-transduction pathways and induce modifications of the tension and the structure of the cytoskeleton as well as changes in the nucleus as a response. Since the nucleus, the nuclear membrane and the chromatin are directly connected to the cytoskeleton, any mechanical stimuli cause changes in the chromatin state thus leading to changes in gene expression [7,43].

Here, cell spreading was strongly reduced 24 h post-seeding on totally laser-nanostructured surfaces (Figure 3). This decrease can be explained based on a hypothesis that our textured samples might work as anti-adherent surfaces. As a result, cells would become less prone to spread. hMSCs cultured on specific patterns inducing cell shape with higher aspect ratios enhance the cytoskeletal tension and exhibit a clear tendency to differentiate towards osteoblastic lineage [25,26,27]. Interestingly, McBeath et al. [28] observed that hMSCs grown on pro-adherent patterns would differentiate into osteoblastic cells or adipocytes if sufficiently spread or unspread, respectively. It seems that adjusting cellular spreading would control stem cell lineage commitment [28]. Dumas et al. [10] showed that modifications of cell morphology induced by laser-nanostructured surfaces could direct MSCs differentiation into osteoblasts.

### 4.2. Multiscale Surface Topography (Laser-Patterned Samples with Polished Surface Areas (LP_LIPSS), and Laser-Patterned Samples with Spikes (LP_spikes))

Time-lapse fluorescence microscopy was used for cell motility measurements, 1 h post-seeding and for 63 h. An increase of the cellular velocity was observed for hMSCs seeded on totally laser-nanostructured samples, but not on laser-patterned ones (relative to polished). Bertolo et al. [44] demonstrated that cell velocity is correlated with cell spreading and that faster cells have small spreading areas. This is adequate for our results. Moreover, the augmentation in cell velocity, acceleration and tortuosity on the totally laser-nanostructured samples denote a tendency for cells to explore their environment. We assume that the exploration of the environment by the cells results from the search for more suitable adherent sites. Interestingly, the velocity and the acceleration of hMSCs seeded on laser-patterned surfaces (LP_LIPSS and LP_spikes) were significantly reduced compared with totally laser-nanostructured samples, and even reached the average velocity measured for hMSCs on polished surfaces (Figure 4b left panel). This could be explained both by the fact that the polished surface may offer more possible adherent sites and that pits can constitute a location for cell implantation. Indeed, it was widely observed that cells slowed down during pit-crossing or even settled down, placing their nucleus inside the pit (Supplementary Materials, Video S2). Further measurements showed that on LP_LIPSS, up to 70% of the cells’ nuclei were located inside pits. This last result proved that the control of the cell “engraftment” could be performed using topography modifications at the microscale. The fact that this behavior might simply be the consequence of gravity was discarded since it was observed during real-time observation with upside-down samples. As shown by Pieuchot et al. [45], cells respond to cell-scale curvature variations during their migration and position themselves in concave valleys such as the pits on LP_LIPSS. It is worth noting that the nuclei were not significantly located inside pits on LP_spikes. This could be explained by the pattern topography itself (Figure 1b) which is better defined in terms of height on LP_LIPSS compared to LP_spikes. Indeed, on LP_LIPSS, only pits represent a concave zone, while on LP_spikes the higher laser fluence used to generate spikes led to slightly deeper areas (−1.5 µm), which could also be considered as concave.

To validate our previous idea connecting cell motility and the research for suitable adherent sites, focal adhesion studies were performed on laser-patterned samples. We showed that hMSCs adhered preferentially to polished areas compared to the nano-textured ones, and this result was enhanced on LP_LIPSS (Figure 6). Since the cells also adhered to totally textured samples, it is hard to conclude whether laser-nanostructured areas had intrinsic anti-adhesive properties. Nevertheless, when grown on laser-patterned surfaces (i.e., a mix of polished and laser-nanostructured areas), a significant amount of the cells placed their focal adhesion points on polished areas rather than on textured ones. Studies indicated that cell adhesion is improved on moderate hydrophilic biomaterial surfaces. Dowling et al. [46] indicated that a surface with a wettability comparable to our smooth titanium (60–80° contact angle) showed better cell adhesion compared to more hydrophilic (12°) or more hydrophobic ones (155°). However, cell adhesion is highly governed by surface properties including roughness, and chemical composition, such complex interrelationships are the possible reasons for the contradictory reported behaviors. For example, superhydrophobic surfaces were reported to be extremely cell repellent [47,48] whereas it was shown, in another work, that cells adhere and proliferate on superhydrophobic surfaces [49]. In our study, we have noted that there were fewer focal contacts on the smooth areas of the LP_spikes compared to the LP_LIPSS (Figure 6), this could be due to the fact that the polished areas are not at the same height level than the areas with nanostructures in LP_spikes. This irregular depth in LP_spikes could decrease the development of focal adhesions on smooth areas. In order to achieve a better understanding of the connection between laser-induced topographies and anti-adherent properties, we studied the protein adsorption rate of such laser-patterned surfaces. The surface wettability was shown to be an important factor in protein adhesion to biomaterial surfaces. Generally hydrophobic surfaces are considered to be more protein-adsorbent than are hydrophilic surfaces [50]. However, different studies regarding the effects of surface wettability on protein adhesion have not always been consistent, the adhesion of proteins to a surface is a time-dependent process that can involve relatively large energy scales [51]. In the present study, it was found that albumin adsorption was significantly higher on polished areas as compared to laser-nanostructured ones. Studies have shown that cells do not adhere directly to the surface but to proteins, in turn adsorbed on the first surface layer [52]. The protein layers formed within the first few minutes of contact with the culture medium consist almost exclusively of albumin [53]. This initial protein adsorption behavior that affect cell-adhesive protein such as fibronectin could subsequently regulate the cell adhesion via focal contacts [54,55]. Therefore, variations in albumin adsorption rate should be representative of favorable focal adhesion locations. These results strongly suggest that within laser-patterned surfaces, the laser-nanostructured areas behaved like anti-adhesive sites for proteins and cells. Therefore, laser patterning could be used to create local “anti-adherent sites” in order to constrain and stimulate the stem cells to form the desired shape. Indeed, in this study, star-shaped cells were observed on laser-patterned surfaces. Polished and pro-adherent branches were guiding lines for cell extensions and the remoteness of adhesion point locations promoted a high contractility of the cell cytoskeleton. Furthermore, it is known that cytoskeleton contractility and star-shaped hMSCs enhance stem cell differentiation into the osteoblastic lineage. Indeed, Kilian et al. [25] demonstrated that changing the shape of the patterns altered the osteogenesis, so stem cells grown on flower-shaped (low contractility) or star-shaped (high contractility) patterns would differentiate into adipocytes or osteoblasts, respectively.

Altogether, our results strongly suggest that surfaces with a predictive cell behavior can be developed from the understanding of cell-surface interactions. Such surfaces will constitute a major improvement for the control of the interface between the implant and the surrounding tissues. Complex multiscale surface geometries (LP_LIPSS and LP_spikes) combining micro-pits and nanostructures lead to an efficient cell positioning on specific textured areas and the control of focal contact adhesion repartition. Creating anti-adherent sites delimits appropriately the adhesive sites elsewhere, and the combination of the two leads to cell confinement for control of shape and mechanics. We expect that mechanically strained cells on a patterned surface should differentiate preferentially into osteoblastic cells. A correlation between late differentiation and early parameters, such as shape or migration, is not within the scope of the present report. A separate study will be carried out on this subject matter.

The laser texturing as a single-step process for creating micron- and nanoscale directly on metals could constitute a potential tool to improve the performance of biomedical titanium and its attributes would be useful for a range of applications in regenerative medicine including orthopedics and dentistry. Otherwise, the anti-adherent function can be applied to achieve other functionalities for other biomedical implants, for instance, the contact surfaces of nails and splints for bone fracture fixation where a minimum cell adherence is highly desired.

## Figures and Tables

**Table 1:** Laser parameters.

Topographies	Pulse Energy (E)	Fluence Peak	Pulse Rate	Distance between Pulses	Hatch Distance	Number of Pass
**Micro-Pits**	32 × 10^−8^ J	0.31 J·cm^−1^	10 kHz	2 µm	4 µm	5
**LIPSS**	32 × 10^−8^ J	0.31 J·cm^−1^	100 kHz	4 µm	4 µm	3
**Spikes**	270 × 10^−8^ J	2.59 J·cm^−1^	100 kHz	4 µm	4 µm	1

The fluence peak is defined as: $F_{peak}=\frac{2\;E}{\pi {\;\omega }_{0}{}^{2}}$.

## Supplementary Materials

The following are available online at https://www.mdpi.com/2079-4991/10/5/864/s1: Video S1: Nanostructured surface wettability before and after sterilization process (droplet 360° rotation not shown). Video S2: A representative time lapse video of cell migration for 25 h on LP_LIPSS (5 frame/s).
